# Cost Comparison From a Patient Perspective for Intracranial Stereotactic Radiation Therapy

**DOI:** 10.1016/j.adro.2021.100816

**Published:** 2021-10-26

**Authors:** Rahul N. Prasad, Vedat O. Yildiz, Tejash Patel, Trevor J. Royce, Joshua D. Palmer

**Affiliations:** a Department of Radiation Oncology at the Arthur G. James Cancer Hospital and Richard J. Solove Research Institute, The Ohio State University Comprehensive Cancer Center, Columbus, Ohio; b Center for Biostatistics, Department of Biomedical Informatics, The Ohio State University, Columbus, Ohio; c Doctor of Osteopathic Medicine/Master of Business Administration Program, Philadelphia College of Osteopathic Medicine, Philadelphia, Pennsylvania; d Department of Radiation Oncology, University of North Carolina at Chapel Hill, Chapel Hill, North Carolina

## Abstract

**Purpose:**

Historically, opaque health care pricing in the US has prevented patients from identifying opportunities to lower costs. Attempting to promote price transparency, the US government recently mandated that hospitals publish prices for all services in a document called a chargemaster. Patients often travel to tertiary centers for intracranial stereotactic radiation therapy (SRT), but cost comparison is complicated by multiple delivery systems and fractionation schemes. We hypothesized that prices published in chargemasters vary widely between SRT techniques and institutions.

**Methods and Materials:**

We obtained chargemasters published online by National Cancer Institute–designated clinical centers. Technical charges for Gamma Knife single-fraction stereotactic radiosurgery (GK), single-fraction linear-accelerator stereotactic radiation surgery (SRS), and 3-fraction fractionated stereotactic radiation therapy (FSRT) were obtained from chargemasters by billing code and keyword searches. Prices were adjusted by the Medicare geographic cost price index (GPCI). Pairwise comparisons were conducted to compare prices between modalities and geographic regions. Relationships with cost index were examined using Spearman correlations, as was the price interrelationship between modalities across institutions.

**Results:**

Of 62 chargemasters obtained, 58 listed SRT prices. Median prices were $49,529 for GK, $31,834 for FSRT, and $22,915 for SRS. Prices varied widely, with large ranges corresponding to 2 to 9 times the magnitude of median prices (GK, $111,298; FSRT, $312,480; and SRS, $104,396). Adjusting for GPCI, GK (*P* = .0003) and FSRT (*P* = .001) were more expensive than SRS, and no difference in price was noted between regions. The FSRT price was positively correlated with GPCI (*P* = .033), but prices for the other techniques were not. Modality prices were all positively correlated (all *P* < .001), meaning that institutions with prices greater than the median price for SRS were similarly expensive for GK and FSRT.

**Conclusions:**

Published prices for SRT vary by delivery system, fractionation, and institution without a clear explanation. Obtaining personalized price estimates may offer cost savings for patients. Policy changes encouraging reliable access to insurer-negotiated cost estimates for SRT are needed.

## Introduction

Patients with cancer are particularly vulnerable to damaging disease and therapy-related financial distress.^1^^,^^2^ Bankruptcy rates for these patients are more than double that of their peers,^3^ and economic hardship is linked to poorer quality of life and cancer outcomes.^4^, ^5^, ^6^, ^7^ Inability to make copayments^8^ is a considerable cause of economic distress, and opaque health care pricing frequently exposes patients to unexpected and crippling out-of-pocket bills.^9^ In 2019, the US Centers for Medicare & Medicaid Services (CMS) required that all US hospitals publish a comprehensive list of charges for all offered services, called a chargemaster,^10^ with the aim that improved price transparency would drive down costs by encouraging price comparison by all stakeholders including patients, insurers, and providers.

Intracranial stereotactic radiation therapy (SRT) is well suited for price comparison, because it is a high-cost, nonemergent, and short-duration (1 to 5 daily treatments) intervention with numerous benign and malignant indications^11^ for which patients are frequently referred to high-volume centers.^12^ Financial toxicity is defined as the financial burden encountered by patients receiving cancer therapy, a broad definition intended to encompass both subjective and objective measures of economic distress.^8^^,^^13^ Whereas the financial toxicity rate of SRT has not been prospectively studied, a rate of early-onset financial toxicity of more than 20% has been noted after definitive radiation therapy, suggesting that patients receiving SRT may be at high risk for therapy-related economic harm.^14^ Multiple referral centers may exist within a city, state, or region, and reliable cost data, if available, could help patients choose a facility that minimizes out-of-pocket costs. However, price comparison is complicated by the presence of multiple delivery systems and fractionation schemes including Gamma Knife or CyberKnife single-fraction stereotactic radiation surgery (GK), single-fraction linear accelerator (linac)–based stereotactic radiosurgery (SRS), and multisession linac-based fractionated stereotactic radiation therapy (FSRT). To our knowledge, a robust analysis of the availability of upfront cost-estimate data for these SRT techniques has not been previously reported, and a systemic head-to-head price comparison has not been attempted. Although the CMS previously equated Medicare reimbursement for GK and SRS (with private payers expected to act similarly),^15^ we predicted that published prices for intracranial SRT listed in chargemaster documents would still differ by delivery system to reflect historical information. We also hypothesized that published prices for intracranial SRT listed in chargemaster documents would vary by fractionation scheme and between institutions.

## Methods and Materials

A total of 63 National Cancer Institute (NCI)–designated cancer centers offering clinical care were identified; 7 laboratory-only sites and St. Jude Children's Research Hospital, which offers free pediatric care only, were excluded. In late 2020, the chargemasters for each institution were obtained through Google-based online search queries using search terms such as “chargemaster,” “charge master,” and “price list.” Only price data acquired from chargemasters were analyzed, as institutions do not provide other sources of price information for intracranial SRT. Because only publicly listed cost data were used, institutional review board approval and informed consent were not necessary.

For each institution, published technical charges for GK, linac SRS, and 3-fraction FSRT delivery were obtained from the chargemaster corresponding to the Current Procedural Terminology (CPT) billing codes 77371 for GK radiation treatment delivery, 77372 for single-fraction linac SRS treatment delivery, and 77373 for multifraction stereotactic therapy. The delivery portion of radiation therapy was assessed because it is a highly standardized charge that captures the majority of the cost of the total price of radiation therapy.^16^ When CPT codes were not available in the chargemaster documents, a manual search using terms such as “SRS,” “stereotactic radiosurgery,” and “cranial” was conducted to identify the appropriate SRT delivery list prices. Additional terms such as “linac” or “linear accelerator” were used to differentiate SRS and terms such as “Gamma Knife,” “Cobalt 60,” “SRS multisource,” and “Cyberknife” were used to identify GK prices. The cost of FSRT was separated out using search terms such as “fractionated” or “per fraction.” In standard fashion, the listed per-fraction cost for each fraction of FSRT was multiplied out to reflect the price for a 3-treatment course unless an institution published an alternate way to estimate the cost, such as a total cost for 2 to 5 fractions of SRT regardless of the number of delivered fractions. Of note, the price for single-fraction SRS was never used to calculate the price for FSRT, because the billing code for multifraction SRT (77373) is a completely distinct entity from the code for SRS.

Institutional cost data were summarized using descriptive statistics with medians and quartiles calculated for continuous variables, because prices followed a nonnormal distribution. We evaluated the difference in published price between GK, SRS, and FSRT across all included institutions after adjusting for the facility-specific reimbursement modifier assigned by Medicare to account for differences in the cost of living, called the practice expense geographic practice cost index (GPCI). We compared the cost for each of the 3 techniques between geographic regions to evaluate regional disparities. In addition, we used logistic regression and Spearman correlations to assess the relationship between GPCI and published prices for each modality to investigate whether differences in price were reflective of reimbursement differences associated with cost of living. Furthermore, we used logistic regression and Spearman correlations to examine the interrelationship between GK, linac SRS, and FSRT prices to assess, for example, whether an elevated published price for one technique correlated with high prices for the others. Spearman correlations were used, because data were not normally distributed. Statistical tests were 2-sided with statistical significance evaluated at the α = 0.05 significance level.

## Results

**Fig. 1:**
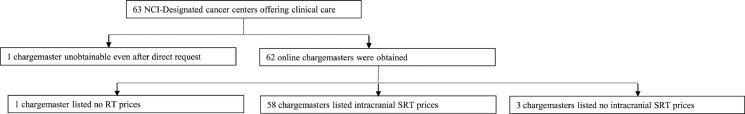
Schematic documenting process for obtaining intracranial stereotactic radiation therapy (SRT) cost data. *Abbreviations:* NCI = National Cancer Institute; CPT = current procedural terminology.

Of the 63 NCI-designated cancer centers offering clinical care, 62 had obtainable chargemasters (Fig 1). These were all easily acquired through an Internet search using 1 or 2 search attempts using the search phrases specified in the methods except for 1 document that was not published and was acquirable only after an email request to the institution. Of note, 1 chargemaster was not downloadable from the institution's webpage without providing an email address. One institution did not provide a chargemaster even after direct email request. Of the 62 chargemasters that were acquired, 1 chargemaster did not list prices for any form of radiation therapy. Three additional chargemasters did not list prices specific to delivery of any form of intracranial SRT. Thus, in total, 58 institutions’ chargemasters were included in the quantitative analysis. Fifty-one (88%) of these chargemasters were downloadable files compatible with Microsoft Excel or Adobe Acrobat. Seven were not downloadable and were searchable by online query only.

**Fig. 2:**
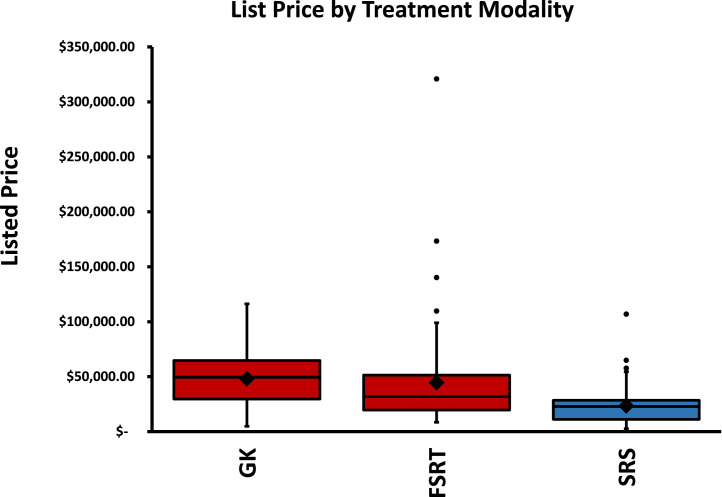
Comparison of published prices for Gamma Knife stereotactic radiosurgery (GK), linear accelerator stereotactic radiosurgery (SRS), and linear accelerator fractionated stereotactic radiation therapy (FSRT) across National Cancer-Institute designated cancer centers. *P* < .0001 after adjusting for cost of living. Line indicates the median; diamond indicates the mean; dots indicate outliers.

Ultimately, 31, 58, and 57 institutions’ listed prices were obtained for GK, SRS, and FSRT, respectively. The median prices for GK, SRS, and FSRT were $49,529, $22,915, and $31,834, respectively (*P* <.0001), and GK and FSRT were both significantly more costly than SRS (*P* = .0003 and *P* = .001, respectively) but not significantly more expensive than each other (*P* = .271). Figure 2 depicts the wide variation in the mean and median prices between institutions for GK, SRS, and FSRT. Owing to the presence of outlier prices for SRS and FSRT, price ranges were large (GK, $111,298; SRS, $104,396; and FSRT, $312,480). Thus, the maximum prices for GK, SRS, and FSRT of $116,208, $107,000, and $321,000, respectively, resulted in differences in maximum and minimum prices by factors of 23.7, 41.1, and 37.7, respectively. When prices for the 10th- and 90th-percentile institutions were compared to mitigate the effect of outliers, prices still differed by factors of 9.4, 7.8, and 6.2, respectively, for GK, SRS, and FSRT (Table 1). These ratios exceed historical, expected rates of variability in reimbursement for outpatient interventions.^16^^,^^17^ Even interquartile ranges (GK, $35,037; SRS, $17,295; and FSRT, $31,743) were equivalent to 71% to 100% of median prices in magnitude, which reflects substantial variability in price between the 25th- and 75th-percentile institutions for each SRT technique.

**Table 1:** Published prices for GK, SRS, and FSRT between institutions in the 10th and 90th percentile

	Published price		90th percentile / 10^th^ percentile
Technique	10th percentile	90th percentile	
GK	$8,269.35	$77,569.20	9.4
SRS	$5,290.62	$41,338.50	7.8
FSRT	$12,392.46	$76,425.60	6.2

*Abbreviations:* FSRT = linear accelerator fractionated stereotactic radiation therapy; GK = Gamma Knife stereotactic radiosurgery; SRS = linear accelerator stereotactic radiosurgery.

*The comparison of published prices for GK, SRS, and FSRT between the 10th- and 90th-percentile institutions eliminates outlier values but still shows substantial price variability, with ratios ranging from 6.2 to 9.4.

**Fig. 3:**
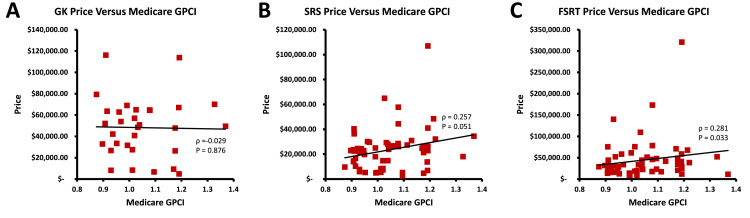
Relationship between cost of living as per the Medicare practice expensive geographic practice cost index (GPCI) and published prices for A, Gamma Knife stereotactic radiosurgery (GK); B, linear accelerator stereotactic radiosurgery (SRS); and C, linear accelerator fractionated stereotactic radiation therapy (FSRT). The FSRT price was positively correlated with GPCI (*P* = .033), but the GK and SRS prices were not (*P* = .876 and 0.051, respectively)

The FSRT price was positively correlated with the GPCI (*P* = .033), but the GK and SRS prices were not (*P* = .876 and 0.051, respectively) (Fig 3**)**. Table 2 shows that all modality prices were interrelated, because all were positively correlated with one another (all *P* < .001). This finding means that, for example, an institution with a price greater than the median for SRS would tend to have a price greater than the median for GK and FSRT as well. There was no adjusted difference in the price for any of the 3 SRT techniques when comparing between different geographic regions (Table 3). However, when examining prices for modalities within the geographic regions, there was a significant difference in price between modalities (*P* = .017) within the East North Central region. In this region, GK was significantly more costly than SRS (*P* = .0042) and FSRT (*P* = .031). Notably, there was no significant price difference between modalities within any of the other regions.

**Table 2:** Relationship between prices for GK, SRS, and FSRT

Technique	GK		SRS		FSRT	
	Spearman rho	*P* value	Spearman rho	*P* value	Spearman rho	*P* value
GK	1	-	0.58	0.0005	0.46	0.0008
SRS	0.58	0.0005	1	-	0.66	<0.0001
FSRT	0.46	0.0008	0.66	<0.0001	1	-

*Abbreviations:* FSRT = linear accelerator fractionated stereotactic radiation therapy; GK = Gamma Knife stereotactic radiosurgery; SRS = linear accelerator stereotactic radiosurgery.

**Table 3:** Price comparison by region after adjusting for cost of living

Region	GK			SRS			FSRT			*P* value
	Median	25th Percentile	75th Percentile	Median	25th Percentile	P75	Median	25th Percentile	75th Percentile	
East North Central	57241.5	41245.7	89935.5	16768	10350	28081	28857	15573	32352	0.017
East South Central	56169.3	32916.7	79422	16308	9659	22957	30405.5	28977	31834	0.101
Mid-Atlantic	64480	42237	67050	26331	22872	48512	49173	29658	78552	0.125
Mountain	55731.6	48602.1	62861	26946	19583.5	29737.5	67008.9	35162.5	92660.4	0.232
New England	4910	4910	4910	23924	6902	27355.1	21728.9	17214	58788	0.318
Pacific	48696.2	26480	64792.3	25643.5	21326	28642	39701	22518	51363	0.086
South Atlantic	39519.5	8243	65000	16024	5619	23908	21643	13918.5	43324.5	0.173
West North Central	27527	8374.74	53978	23059	8374.74	29654	26606.3	18498	33816	0.741
West South Central	48934	40819	57049	8514	7361	14779	19620	8520	44337	0.102
*P* value	0.16			0.398			0.351			

*Abbreviations:* FSRT = linear accelerator fractionated stereotactic radiation therapy; GK = Gamma Knife stereotactic radiosurgery; SRS = linear accelerator stereotactic radiosurgery.

## Discussion

This analysis of published prices obtained from publicly available, online chargemasters from NCI-designated cancer centers suggests that despite overlapping indications, the listed technical charges, adjusted for cost of living, for GK and FSRT are higher than for SRS. Even after excluding outlier pricing, estimates from high-cost institutions are as much as 6 to 9 times higher than quotes from other high-volume academic institutions that are less expensively priced. These findings are noteworthy, because to our knowledge, a systemic comparison of the published prices between these treatment techniques and between institutions has not been previously reported. Economic distress after cancer therapy is already highly prevalent,^1^^,^^2^ occurs early after radiation therapy,^14^ and is associated with inferior outcomes.^4^, ^5^, ^6^, ^7^ Potential cost differences for intracranial SRT modalities between treatment facilities are becomingly increasingly meaningful, because owing to advances in systemic therapy, patients with cancer live longer and are more prone to developing brain metastases.^18^ Insurers may absorb a significant portion of potentially avoidable upfront costs resultant from therapy. However, the financial consequences are ultimately borne by patients in the form of increased deductibles, premiums, or copayments.

By encouraging cost competition by all stakeholders (including patients, providers, and insurance companies), improved price transparency is believed to be one relatively concrete and achievable way to lessen costs.^9^ Rules promoting price transparency not only allow patients to price compare between facilities but also help payers leverage competitors’ price data to negotiate favorable prices with providers.^9^ Price comparison makes particular sense for expensive, nonurgent, and brief episodes of care such as SRT. In 2019, to try to improve price transparency, the CMS decreed that US hospitals must publish a consumer-friendly list of standard charges for all items and services in a machine-readable document called a chargemaster.^10^ Starting on January 1, 2021, the CMS additionally mandated disclosure of insurer-negotiated prices for services in a “shoppable,” user-friendly format, with emphasis placed on the presentation of 300 common services.^19^ However, owing to the limitations of this regulation, analysis of the prices for intracranial SRT published in chargemasters may actually be more relevant. Although rates of compliance with the mandate to provide standard charges are quite high, rates of reporting of negotiated rates are quite low, including at the 100 hospitals with the highest revenue, a group that encompasses many NCI-designated centers.^20^ In this context, analysis of data derived from the NCI-designated institutions providing payer-negotiated rates for intracranial SRT alone would be subject to substantial reporting bias; the institutions that do and do not report negotiated data may differ significantly. Because the mandate to include 300 common services in a “shoppable” format is not inclusive of prices for any oncology service including any form of radiation therapy,^19^ rates of reporting negotiated prices for intracranial SRT are likely to be even lower. For these reasons, the precise effect of insurer negotiations on prices for SRT is not assessable. Our analysis showed extreme price variability in line with previously reported analyses^16^^,^^21^ and expands on them by noting that cost variation between institutions is replicated across the comparable techniques of SRS and FSRT. Some difference in prices between institutions is highly likely to persist even after negotiation by insurers, given the magnitude of the differences we found and that these differences were borne out over a large number of high-volume institutions nationwide. Notably, even negotiated prices vary widely between institutions, as insurers routinely negotiate entirely different costs for the same service for patients at multiple institutions depending on factors such as relative bargaining power that are not necessarily tied to clinical outcomes.^17^ In this context, our findings provide evidence that patients interested in cost control should obtain price estimates negotiated by their health insurance company from multiple institutions.

The difference in self-reported, linear-accelerator SRS and FSRT prices may relate to increased use of resources. Although the treatment simulation and planning process is similar, treatment over multiple days instead of 1 session involves additional physics, physician, treatment machine, and therapist resources. The significantly increased technical charges for GK versus SRS likely reflect historical differences in reimbursement, because the CMS previously equalized Medicare reimbursement for GK and SRS and private insurers were expected to follow suit.^15^ If so, these estimates are highly misleading and potentially damaging to patients.

We also found that prices for all 3 SRT techniques were interrelated, meaning that an institution priced expensively for 1 modality tended to also have high prices for the other techniques. This result may reflect tendencies by institutions to have similarly high or low prices for services either across all service lines or within radiation oncology. We also found that price variation for single-fraction radiation surgery is poorly explained by cost of living, regardless of delivery system, and that price variation for FSRT is only weakly explained by GPCI. We also found no significant difference in cost between geographic regions for any modality. Overall, these findings suggest that pricing may be largely arbitrary, but comparing costs within a reasonably sized geographic region may be sufficient as opposed to traveling farther for cost savings.

### Study limitations

Limitations of this analysis primarily stem from the limits of publicly available data. An analysis of copayments for intracranial SRT by modality and between institutions would be helpful, but hospitals are not required to provide this information and it is not available in any standardized fashion. Of note, evaluating copayments alone limits generalizability to certain patient populations such as the uninsured or patients from overseas for whom standard prices are applicable. Next, we analyzed the technical fees for radiation delivery, which omits a portion of the total cost of therapy.^16^^,^^22^ Accounting for nontechnical charges in a standardized fashion was not feasible owing to the absence of uniform, nontechnical charge packages and terminology between institutions. Focusing on technical charges may be the most direct measure of price inconsistency. An in-depth cost-effectiveness study is another way to evaluate cost differences between SRT delivery methods but is less generalizable because it is not possible to collect proprietary data from more than a few institutions; this approach therefore incompletely answers the question of cost comparison between institutions. Lastly, we only analyzed price data from NCI-designated centers, which may limit generalizability to lower-volume institutions; however, these referral centers treat a disproportionately high volume of SRT patients.

## Conclusions

Published prices for intracranial SRT vary by delivery system and fractionation and between institutions with little explanation, and they also have limited correlation to Medicare reimbursement modifiers tied to the cost of living. This first-in-kind analysis generates relevant hypotheses for ways to decrease costs for patients, most importantly by encouraging patients to obtain multiple price estimates, preferably tailored to their insurance plan and clinical situation. Policy changes that standardize base pricing or provide insurer-negotiated estimates for radiation therapy would greatly benefit patients.

## References

[1] Altice CK, Banegas MP, Tucker-Seeley RD, Yabroff KR. (2017). Financial hardships experienced by cancer survivors: A systematic review. J Natl Cancer Inst.

[2] Gordon LG, Merollini KMD, Lowe A, Chan RJ. (2017). A systematic review of financial toxicity among cancer survivors: We can't pay the co-pay. Patient.

[3] Ramsey S, Blough D, Kirchhoff A (2013). Washington State cancer patients found to be at greater risk for bankruptcy than people without a cancer diagnosis. Health Affairs.

[4] Carrera PM, Kantarjian HM, Blinder VS. (2018). The financial burden and distress of patients with cancer: Understanding and stepping-up action on the financial toxicity of cancer treatment. CA Cancer J Clin.

[5] Fenn KM, Evans SB, McCorkle R (2014). Impact of financial burden of cancer on survivors’ quality of life. J Oncol Pract.

[6] Hazell SZ, Fu W, Hu C (2020). Financial toxicity in lung cancer: An assessment of magnitude, perception, and impact on quality of life. Ann Oncol.

[7] Ramsey SD, Bansal A, Fedorenko CR (2016). Financial insolvency as a risk factor for early mortality among patients with cancer. J Clin Oncol.

[8] Zafar SY, Peppercorn JM, Schrag D (2013). The financial toxicity of cancer treatment: A pilot study assessing out-of-pocket expenses and the insured cancer patient's experience. Oncologist.

[9] Henrikson NB, Shankaran V. (2016). Improving price transparency in cancer care. JOP.

[10] Centers for Medicare & Medicaid Services. Frequently asked questions regarding requirements for hospitals to make public a list of their standard charges via the internet. Available at: https://www.cms.gov/medicare/medicare-fee-for-service-payment/acuteinpatientpps/downloads/faqs-req-hospital-public-list-standard-charges.pdf. Accessed February 1, 2021.

[11] Gilbo P, Zhang I, Knisely J. (2017). Stereotactic radiosurgery of the brain: A review of common indications. Chin Clin Oncol.

[12] Solberg TD, Balter JM, Benedict SH (2012). Quality and safety considerations in stereotactic radiosurgery and stereotactic body radiation therapy: Executive summary. Pract Radiat Oncol.

[13] Schroeder SR, Agusala V, Sher DJ. (2019). Financial toxicity and cancer therapy: A primer for radiation oncologists. Hematol Oncol Clin North Am.

[14] Palmer JD, Patel TT, Eldredge-Hindy H (2018). Patients undergoing radiation therapy are at risk of financial toxicity: A patient-based prospective survey study. Int J Radiat Oncol Biol Phys.

[15] McClelland S, Degnin C, Chen Y, Watson GA, Jaboin JJ. (2019). Impact of the American Tax Payer Relief Act on stereotactic radiosurgery utilization in the United States. J Neurooncol.

[16] Agarwal A, Dayal A, Kircher SM, Chen RC, Royce TJ. (2020). Analysis of price transparency via National Cancer Institute—Designated cancer centers’ chargemasters for prostate cancer radiation therapy. JAMA Oncol.

[17] Koller CF, Khullar D. (2019). The commercial differential for hospital prices: Responses from states and employers. JAMA.

[18] Jablonska PA, Bosch-Barrera J, Serrano D, Valiente M, Calvo A, Aristu J. (2021). Challenges and novel opportunities of radiation therapy for brain metastases in non-small cell lung cancer. Cancers (Basel).

[19] Centers for Medicare & Medicaid Services. Hospital Price Transparency Frequently Asked Questions. Available at: https://www.google.com/search?q=Hospital+Price+Transparency+Frequently+Asked+Questions&rlz=1C5CHFA_enUS806US806&oq=Hospital+Price+Transparency+Frequently+Asked+Questions&aqs=chrome.0.69i59j69i60.318j0j4&sourceid=chrome&ie=UTF-8. Accessed February 1, 2021.

[20] Gondi S, Beckman AL, Ofoje AA, Hinkes P, McWilliams JM. (2021). Early hospital compliance with federal requirements for price transparency. JAMA Intern Med.

[21] Xiao R, Miller LE, Workman AD, Bartholomew RA, Xu LJ, Rathi VK. (2021). Analysis of price transparency for oncologic surgery among National Cancer Institute–designated cancer centers in 2020. JAMA Surg.

[22] Moore A, Stav I, Den RB (2019, January 2). The financial impact of hypofractionated radiation for localized prostate cancer in the United States. J Oncol.

